# Fat Embolism Syndrome – A Qualitative Review of its Incidence, Presentation, Pathogenesis and Management

**DOI:** 10.5704/MOJ.2103.001

**Published:** 2021-03

**Authors:** C Timon, C Keady, CG Murphy

**Affiliations:** Department of Trauma and Orthopaedics, Galway University Hospitals, Galway, Ireland

**Keywords:** fat embolism syndrome, fat embolism, trauma, orthopaedics

## Abstract

Fat Embolism Syndrome (FES) is a poorly defined clinical phenomenon which has been attributed to fat emboli entering the circulation. It is common, and its clinical presentation may be either subtle or dramatic and life threatening. This is a review of the history, causes, pathophysiology, presentation, diagnosis and management of FES. FES mostly occurs secondary to orthopaedic trauma; it is less frequently associated with other traumatic and atraumatic conditions. There is no single test for diagnosing FES. Diagnosis of FES is often missed due to its subclinical presentation and/or confounding injuries in more severely injured patients. FES is most frequently diagnosed using the Gurd and Wilson criteria, like its rivals it is not clinically validated. Although FES is a multi-system condition, its effects in the lung, brain, cardiovascular system and skin cause most morbidity. FES is mostly a self-limiting condition and treatment is supportive in nature. Many treatments have been trialled, most notably corticosteroids and heparin, however no validated treatment has been established.

## Method

SRQR guidelines were employed to guarantee the transparency of this qualitative research. This formula defines a clear standard for reporting qualitative research while preserving the requisite flexibility to accommodate various paradigms, approaches and methods. This qualitative systematic review provides an up-to-date summary of the incidence, presentation, pathogenesis and management of Fat Embolism Syndrome.

## Definition and Introduction

Fat embolism^1^ occurs when fat enters the circulation, this fat can embolise and may or may not produce clinical manifestations.

FES is a poorly defined clinical phenomenon which has been attributed to fat emboli entering the circulation. It classically presents with respiratory, neurological and dermatological features. It typically occurs after long-bone fractures and total hip arthroplasty, less frequently it is caused by burns and soft tissue injuries^2^. Early stabilisation of long bone fractures is thought to reduce its incidence, however the most effective means of achieving this has yet to be determined^3^. It is one of the least understood complications of trauma.

## Epidemiology and Incidence

FES occurs most frequently following orthopaedic trauma; however, it has been documented in the literature as having occurred following other traumatic conditions such as burns, hepatic injury, cardiopulmonary resuscitation compressions, bone-marrow transplant, lung transplant, liposuction, caisson disease, extracorporeal circulation, tetrachloromethane poisoning, and caesarean section. Fat emulsion injection, corticosteroid therapy, pancreatitis, angiomyolipoma, intravenous lipid infusion, and haemoglobinopathies such as sickle cell disease (SCD) are rare non-traumatic causes of FES^4-8^.

Incidence of fat embolism and FES varies significantly throughout the literature depending on series design. Long-term retrospective review reports the lowest rates. Muller reported the incidence of fat embolism in long bone fractures is 0.9% to 2.2% of cases^9^ and that during intramedullary manipulation/ instrumentation the incidence tends to be lower (0.5 – 0.8%).

Prospective studies report far higher yet consistent rates of FES, while the incidence of FE at autopsy is significantly greater than the incidence of clinically suspected FES^9^.

FES occurring after cosmetic procedures such as liposuction and gluteal vitamin E injections is a well-described phenomenon that has even led to fatalities^10,11^. While these aesthetic procedures are relatively safe, clinicians should be aware of the possibility of FES in the postoperative period, and exercise suspicion should their patient become acutely unwell.

FES due to extensive bone marrow necrosis in Sickle Cell Disease (SCD) is also a rare but well-described complication with a mortality rate of 64%^12^. Surprisingly, patients with the “milder” form of SCD seem to be most at risk, 81% of patients in a review article had a genotype other than HbSS.

### Incidence diagnosed by clinical criteria

Gurd, in his 1974 paper reported the incidence of FES as 19% in a group of trauma patients^5^. In a 10-year review carried out by a major American trauma centre an incidence of 0.9% was reported^13^. Interestingly, the authors found no correlation between the severity, location or pattern of patients' injuries and their likelihood of developing FES, contradicting other studies which have shown a significant association between “at-risk fractures” (fractures of the femur, tibial shaft or pelvis) and incidence of FES^14^. The same study also reported a correlation between the number of fracture sites and the incidence of FES.

### Incidence established by serial monitoring

One trauma centre ran a prospective evaluation where they sought to determine the true incidence of FES following long bone or pelvic fracture. They identified patients with pulmonary shunts by measuring the alveolar/arterial PO2 gradient, and then subcategorised them further to establish a minimum incidence of FES. 49 out of 92 patients had a pulmonary shunt, 39 of them also had a pulmonary injury which was at least partially responsible for the shunt, leading them to conclude that at least 10 patients (11%) had FES secondary to a long bone or pelvic fracture^15^.

### Incidence established by laboratory testing

One series in an arthroplasty centre took serial arterial and right atrial blood samples for histopathology in patients undergoing both unilateral and simultaneous bilateral total knee arthroplasty (TKA). The researchers looked for fat cells at multiple time points and found an incidence of fat embolism of 46% in 100 patients with a unilateral TKA and 65% in 100 patients with bilateral TKAs^16^. Neurological manifestations consistent with FES were reported in two patients from the unilateral group and four patients from the bilateral group Table I.

**Table I:** The incidence and mortality of fat embolism syndrome in recent and landmark series

First Author	Year	Study Design	Incidence(n)	Mortality(n)
Incidence from clinical series				
Robert	1993	25 years retrospective review	0.26% (20)	20% (4)
Bulger EM	1997	10-year review of trauma cases	0.9% (27)	7% (2)
Data from prospective studies				
Schonfeld	1983	Randomised trial of corticosteroids in prophylaxis of FES	15% (9) cumulatively	0
Chan	1984	80 consecutive trauma patients	8.5% (7) 35% of patients with multiple injuries	2.5% (2)
Lindeque	1987	Randomised trial of methylprednisolone’s efficacy	13% (7) by Gird criteria 29% (16) by own criteria	0
Kallenbach	1987	Randomised trial of corticosteroids as prophylaxis in 84 trauma patients	13% (11) cumulatively	Nil
Fabian	1990	96 consecutive long bone fractures	11% (10)	10% (1)
Laboratory testing of blood samples				
Kim YH	2001	Histological staining of arterial and right-atrial blood from intra-operative arthroplasty patients for fat cells	65 (65%) (Bilateral TKA) 46 (46%) (Unilateral TKA)	
Incidence from TOE series				
Pell	1993	Study of 24 tibial and femoral fractures	6(25%) (Significant showers small emboli) 4 (16.6%) (Large emboli) 3 (12.5 %) FES	1 (4.1%)
Christie	1995	111 invasive intramedullary procedures	97 (87%) (Emboli seen)	

Abbreviations: TKA: Total Knee Arthroplasty, FES: Fat embolism syndrome

## Presentation and Clinical Sequelae

FES is both a clinical diagnosis and a diagnosis of exclusion because it is often associated with concomitant injury/ illness. This also leads to difficulty in evaluating the severity of FES.

Signs and symptoms of FES usually present within 24-48 hours of trauma. FES classically presents with changes affecting the respiratory, neurological and dermatological systems.

FES may also present more severely with pulmonary and systemic embolisation of fat, right ventricular failure and cardiovascular collapse. Pell recounted a most dramatic case of fulminant FES that occurred intra-operatively, a patient suddenly developed cor pulmonale during open fixation of a femoral fracture, a patent foramen ovale was discovered at autopsy and a posthumous diagnosis of FES was made^17^.

### Respiratory

Respiratory signs and symptoms are the most frequent presentation of FES. This can range from transient respiratory distress to fulminant respiratory failure. In Lindeque’s study, 16 of 28 patients with lower limb long bone fractures had a PaO2 of less than 7.3kPa^7^, a similar study of polytrauma patients reported a PaO2 of less than 9.3kPa in 90% of included patients. Bulger conducted a 10-year study in a major trauma centre in an attempt to better define FES and diagnosed 27 patients with FES, 12 (44%) required mechanical ventilation12. Diagnosis seems linked to the intensity of focus on signs or symptoms of the syndrome.

### Neurological

Neurological sequelae are also common in FES and presentation can vary dramatically, from mild confusion to drowsiness to a comatose state. DeFroda published a case report of cerebral FES in 2016 of an otherwise healthy young woman presenting with devastating symptoms post reamed intramedullary nail fixation of a femoral fracture^18^. Rare cases of massive cerebral fat embolism leading to brain death are reported^19^. In 2015, a case report was published concerning a woman who died after developing non-convulsive status epilepticus after undergoing total knee arthroplasty. She remained in refractory status epilepticus for two weeks before passing away. At autopsy multiple cerebral infarcts associated with fat emboli were found^20^. This was the first described case of this kind, yet another example of our lack of understanding of the pathophysiology of FES. Scopa described an atypical presentation of FES where neurological symptoms were present without respiratory symptoms^21^. Bulger found that neurological changes were present in 59% of patients^13^. Minor global dysfunction is the most common presentation but there have been dramatic cases of rapid onset hemiparesis, blindness and seizures. Fortunately, neurological symptoms of FES often resolve spontaneously without sequelae^22^. In patients with long bone fractures, who deteriorate neurologically without respiratory compromise, cerebral fat embolism should be suspected, except for those with intracranial lesions, as early diagnosis and comprehensive management can improve prognosis^23^. Magnetic resonance imaging is the imaging modality of choice for cerebral FES^24^.

### Dermatological

A petechial rash is characteristic of FES. This rash typically can be seen on the head, neck, anterior thorax and axillae. Tachakra’s hypothesis is that this pattern is caused by fat droplets aggregating in the aortic arch and then embolising to nondependent skin via the subclavian and carotid vessels25. Stasis, depletion of clotting factors, thrombocytopenia and damage to capillary endothelium from FFAs are all contributory causes of petechial rashes that occur in FES^26^.

### Retinal

Ocular fat embolism syndrome in the absence of cardiac defects is rare but has been described in a case report. The author’s hypothesis was that small fat droplets may pass through the lung capillaries or pre-capillary shunts and enter the systemic circulation^27^. Ocular FES is a separate entity to Purtscher’s retinopathy which is associated with head/chest trauma or acute pancreatitis^28^.

### Laboratory Tests

Laboratory Tests have been used in identifying FES, unfortunately they are non-specific and cannot be reliably used as FES nearly always occurs in the presence of multiple injuries/illness.

Unexplained anaemia occurs in 67% of patients with FES, thrombocytopenia is less prevalent, but still common^13^. The mechanism of this thrombocytopenia is still not understood, platelet consumption due to disseminated intravascular coagulation and platelet activation by bone marrow emboli with thrombus formation are two theories that Riseborough proposed and are yet to be usurped^29^.

Serum phospholipase A2 (sPLA2) is an important inflammatory mediator and liberates FFAs, which are responsible for acute lung injury in FES. Acute Chest Syndrome (ACS) is the leading cause of death in sickle cell disease, in severe ACS fat embolism is often present. It was shown that sPLA2 levels are dramatically elevated in patients with ACS and that levels even correlated with the severity of ACS^30^. Thus, it was proposed that sPLA2 levels could be used to diagnose FES in high-risk patients. Unfortunately, these elevated sPLA2 levels were shown to not be specific to FES and may just be a manifestation of altered lipid metabolism after trauma^31^.

Bronchoscopy and bronchoalveolar lavage (BAL) have also been investigated as means of diagnosing FES^32-34^. Macrophages cells which clean up debris in the lungs at a cellular level could be expected to contain fat cells. Unfortunately, obtaining usable samples proved more difficult than initially thought. In one study 67 out of 96 samples were void due to low yield of macrophages^35^. Furthermore, the stain used to test for fat cells in these investigations (oil red O), stains neutral fat, not FFAs i.e. the fat cells which do not cause acute lung injury^36^. Nevertheless, it is thought that the use of a threshold value (e.g. 30%) of macrophages staining positive for fat could be useful in trauma patients, perhaps even to rule out FES and prompt investigations for an alternative source of hypoxaemia^37^.

### Radiology

Imaging could play a role in FES when neurological involvement is suspected. In the acute setting computed tomography shows no abnormalities^38^, however magnetic resonance imaging (MRI) of the brain has been shown to be useful in early diagnosis of cerebral fat embolism, high-intensity signals are visible in T_2_ weighted images as soon as four hours after symptoms onset. Furthermore, MRI images appear to correlate with the severity of symptoms and with resolution^39^, making MRI as a useful potential tool for grading the severity of cerebral FES and its evolution/resolution.

Suzuki described the lesions as being characteristically along the boundary zones of the major vascular territories and suggested a link between the hypoxic brain condition in FES and the mechanical aggregation of fat globules and blockage of entire brain capillaries^39^. These characteristic findings can be useful for differentiating FES from primary intra-axial brain injury in patients with polytrauma^40^.

More recently an American systematic review was published that identified five distinctive MRI image patterns through the three phases of cerebral fat embolism^41^: scattered cytotoxic oedema occurred in the acute stage, while confluent cytotoxic oedema or vasogenic oedema occurred in the subacute stage, in the long-term brain atrophy and demyelination was identified. Petechial haemorrhages in a confluent shape were seen in all stages of cerebral FES. Early recognition of these patterns may help identify the appropriate management.

In patients with respiratory compromise following trauma or an orthopaedic procedure, diffuse, well-demarcated ground glass opacities or ill-defined centrilobular nodules on computed tomography while non-specific, should give clinicians a cause for suspecting FES^42^. It is vital that radiologists, orthopaedic surgeons and clinicians are familiar with the clinical presentation and imaging of FES to allow for early diagnosis and adequate management.

## Diagnostic Criteria

Fat embolism is a pathological process where fat cells enter the circulation; thus, this phenomenon can only be demonstrated conclusively/empirically at autopsy. FES is the clinical manifestation of this process. There is no single laboratory or radiological test that can make a diagnosis of FES. Several criteria have been designed to diagnose FES. Gurd and Wilson criteria Table II is the most widespread of three criteria proposed to suggest a diagnosis of FES. Gurd proposed his original criteria in 1970 before modifying it with Wilson in 19744,5. A common criticism of Gurd’s criteria is that fat macroglobulinemia is often found in the blood of healthy volunteers and trauma patients with no other evidence of FES^43^.

**Table II:** Gurd and Wilson Criteria for FES^3^

Two major criteria / one major criteria and four minor criteria suggest a diagnosis of FES	
Major Criteria	Minor Criteria
Respiratory Distress Cerebral symptoms in non-head injury patients Petechial rash Renal Changes Retinal Changes Drop in haemoglobin New onset thrombocytopaenia Elevated erythrocyte sedimentation rate Fat macroglobulinemia	Tachycardia (>110 bpm) Fever (>38.5°C) Jaundice

Historically, Schoenfeld’s Table III and Lindeque’s criteria Table IV have also been used. Lindeque *et al*^7^ utilisation of their parameters on this led to a reported incidence of 29%, higher than in any other comparable series. None of the above are clinically validated diagnostic criteria, nor are they accepted universally. Another criticism is that these criteria are overly broad and will capture many non-specific cases of respiratory failure, this may be responsible for the wide variability in the incidence noted in the prior section.

**Table III:** Schonfeld’s scoring system for diagnosing FES^6^

Score >5 points diagnoses FES
Sign/ Symptom: Petechial Rash (5 points) Diffuse infiltrates on chest x-ray chest (4) Fever (1) Tachycardia (1) Tachycardia (1) Confusion (1)

**Table IV:** Lindeque’s Criteria for FES^5^

Criteria:
pO2 <8kpa pCO2 >7.3kpa Respiratory rate >35/min; despite sedation Dyspnoea, tachycardia, anxiety

## Pathophysiology

The pathophysiology of FES remains unclear. The most widely accepted theories are approaching 100 years of age.

### Mechanical theory:

In 1924 Gauss proposed that fat cells from the bone marrow could access venous sinusoids because of the increased intramedullary pressure that occurs following trauma^44^. These adipose cells have proinflammatory and prothrombotic attributes. As they traverse the venous system back towards the heart, they stimulate rapid platelet adhesion and increased fibrin generation, before eventually becoming stuck somewhere in the pulmonary arterial circulation as the vessels form capillaries. Capillary obstruction triggers interstitial haemorrhage, oedema, alveolar collapse and a reactive hypoxemic vasoconstriction. Massive fat emboli may also lead to macrovascular obstruction and shock^45^. Fat cells may enter the arterial circulation via a patent foramen ovale or directly through the pulmonary capillaries causing FES’ characteristic dermatological and neurological findings^46^.

### Biochemical theory:

Lehman hypothesised that the clinical manifestations of FES are caused by a proinflammatory state^47^. Adipose cells from bone marrow are broken down by tissue lipases forming glycerol and toxic free fatty acids (FFAs) e. g., chylomicrons causing injury to pneumocytes and pulmonary endothelial triggering a pro-inflammatory cytokine cascade which can culminate in Acute Respiratory Distress Syndrome. Lehman’s theory may help to explain non-traumatic incidents of FES. Biochemical studies on animal models of FES support this theory.

The toxic properties of FFAs were first demonstrated in the 1950s by Peltier48. Since then FFA infusions have been used in animal-models to induce FES like changes in the circulation and lung^49^. There has been research using triolein, a FFA, to induce FES and investigate a “second hit” phenomenon in rat models. Lung damage following the injection of a pulmonary toxin was worse in rats who had a history of clinically resolved triolein-induced FES than in rats that were exposed to a pulmonary toxin alone^50^.

Aquaporins are cell membrane proteins that form pores in the membrane of biological cells^51^. The expression of an aquaporin called AQP1 was shown by Zhang *et al* to be increased in pulmonary oedema caused by FES^52^. Furthermore, this occurred proportionally to the severity of pulmonary oedema. Zhang proposed that AQP1 could be regulated by FFAs, and therefore upregulated during FES, raising a potential opportunity to target AQP1 as a therapy for FES.

Oleic acid, the most abundant FFA in human adipose tissue^53^, is found in elevated levels in plasma and bronchoalveolar lavage fluids of patients with ARDS^54^. In animal models, it has been shown to provoke alveolar oedema, formation and prevent resolution, thereby contributing to the formation of ARDS^54^. Human albumin has multiple binding sites for FFAs^55^. Stemming from this is the theory that hypoalbuminemia is a predisposing factor for FES in trauma^56^, and it contributes towards an increased mortality risk in hospitalised patients^57^.

There is no clear evidence for the superiority of colloids over isotonic crystalloids in fluid resuscitation, and crystalloid resuscitation has been associated with a lower mortality rate in trauma patients, although the data from this study was not powered enough to influence clinical recommendations^58^. There is divergent data to suggest that, specifically, in the development of ARDS, fluid resuscitation with albumin solutions may be beneficial, because the oleic acid will bind to albumin reducing its potential to induce oedema^59-61^. Furthermore, the use of albumin when combined with furosemide in patients with ARDS tends to improve oxygenation and may reduce length of ventilation60.

## Management

### Pharmacological modalities

Currently there is no specific treatment for FES. A recent animal study showed that aliskiren, the renin inhibitor, protected the lungs of rats from gross and histopathologic damage after FE was induced by injecting fat intravenously^62^. Further studies are needed to see whether aliskiren could be used both, prophylactically before certain orthopaedic operations, and therapeutically after severe trauma to prevent the respiratory consequences of FES. A similar animal study was carried out using losartan on triolein-induced rat models of FES^63^. This study showed us that losartan helped protect the pulmonary system from chronic damage after FES. It also protected the rats from the second phenomenon as mentioned above. Both studies support the possibility of exploiting the renin-angiotensin system in the treatment of FES. Management of FES is often complicated by concomitant injuries and illnesses.

In the mid-20th century treatments such as heparin and dextran were trialled but neither yielded evidence of an improvement in morbidity or mortality^64,65^. As there is no specific treatment for FES, its management if focused on supportive care, symptom control and most importantly prevention.

### Philosophies of Orthopaedic Trauma

Use of corticosteroids in FES prevention is controversial; although, there is evidence that corticosteroids can be used prophylactically to prevent FES in high-risk patients e.g. those who have suffered long-bone fractures^8,66^. However, a 2009 meta-analysis showed that while they did reduce rates of FES and hypoxia, corticosteroids did not reduce mortality rates^67^, and the authors concluded that a larger randomised trial is needed. These studies cannot be applied to modern trauma populations given the ongoing debate about the timing of surgery for long bone fracture has evolved only recently to reach a consensus between Early Total Care and Damage Control Orthopaedics, an approach termed Early Appropriate Care^68^. Furthermore, none of these randomised controlled trials investigated the long-term complications of steroid treatment. Ultimately, a large confirmatory randomised-controlled trial will be needed to allow best practise of evidence-based medicine.

Even though fixation of long bone fractures is the most common cause of FES, prompt and adequate immobilisation of fractures may be very important in preventing the release of further adipose cells into the circulation^69^. Tanton proposed that proper immobilisation of a fracture may help reduce the rates of FES as far back as 1914^70^. A large Finnish trauma centre noticed a trend in decreased rates of FES following a change in policy to fix long bone fractures in the first days after injury^71^. A prospective American study showed that long bone fracture fixation within 24 hours reduced rates of FES, as well as ARDS and pneumonia in patients fit enough for surgery^72^. In fact, delayed fracture fixation has been shown to lead to longer ICU admissions^73^. Pape’s 10-year retrospective study reported that early fracture fixation of a patient with co-existing chest injury led to increased incidence of FES^69^, therefore he proposed that patients with chest injuries should undergo un-reamed intramedullary nailing. This contradicts Brundage’s findings, who concluded that delay of fixation surgery in patients with polytrauma was shown to lead to increased rates of FES as well as other complications and concluded that polytrauma is not a contraindication to early fixation^74^, Pinney *et al* also shared similar findings^75^.

### Fat Embolism During Intramedullary Nailing

The systemic effects of intramedullary nailing were first described by Kuntscher^76^. Increased IM pressure has been shown to cause increased fat embolisation in vitro^77^. Richards found that reamed intramedullary nails were a risk factor for cognitive impairment one year post-operatively^78^. Applying a vacuum or venting during reaming has been shown to lessen intra-medullary pressure and therefore reduce fat embolisation^78^. Volgas monitored intracardiac fatty emboli with intraoperative transoesophageal echocardiography, comparing standard sequential reaming technique with a Reamer Irrigator-Aspirator system. He showed significantly lower levels of fatty emboli in the RIA group^79^. Unfortunately, the significant cost and bulkiness of the RIA system could hinder its widespread adoption. Baig describes the use of a technique which could be used with conventional reaming equipment where the suction tubing for intramedullary reaming is advanced to the end of IM bone and suction is applied for two to three minutes once access to the medullary canal is gained, the procedure is then carried out and suction repeated in a similar manner afterward^80^. Medullary cavity irrigation has also been shown to lead to reduced emboli size on transoesophageal echocardiography during TKA^81^.

Muller compared intramedullary pressure across different reamer systems and found that decreasing the diameter of the flexible driver from 9mm to 7mm significantly reduced pressures. He also reported that using a 9.5mm hollow reamer with a 7mm diameter driver reduced operative intramedullary pressures by 61% to 66%^82^.

In conclusion advances in technique and technology for commonly used reamer systems has facilitated the more aggressive fixation of fractures with reduced likelihood of fat embolisation and the development of FES.

### Management of Concomitant Lung Injuries

ARDS leading to respiratory failure and even death is the most serious potential complication of FES. Management of ARDS secondary to FES aims to maintain adequate gas exchange while minimising the potential for ventilator-associated lung injury (VALI). Enhancement of spontaneous breathing and coughing, early mobilisation, positive end-expiratory pressure (PEEP) and reduced sedation and neuromuscular blockade are some of the supportive therapies used to achieve this^83^. It is also important to consider the overlap of the immunological conditions associated with trauma, and how the impact of FES has not yet been clearly defined within this debate^84^.

Care of patients with FES and neurological complications focuses on frequent neurological observations. Patients with FES may develop cerebral oedema, while this often resolves it sometimes causes severe morbidity^85^. In such cases intra-cranial pressure monitoring should be considered^86^. While clinical diagnosis is still seen as the preferable diagnostic method for FES^87^, studies have shown that MRI is useful, in cases where head trauma is not present in evaluating the severity of cerebral fat embolism and predicting long-term outcomes^24^. In general, sedation and neuromuscular blockade for trauma patients must be titrated so that the patient is kept comfortable, without affecting their serial neurological examinations and, also allowing them to tolerate mechanical ventilation^88^.

## Summary

FES is a poorly defined clinical phenomenon which occurs because of fat emboli entering the circulation. FES mostly occurs secondary to orthopaedic trauma; it is less frequently associated with other traumatic and atraumatic conditions. There is no single test that can be used to diagnose FES. Diagnosis of FES is often missed due to its subclinical presentation or confounding injuries in more severely injured patients. With supportive care the mortality rate is less than 10%, and generally the complete resolution of pulmonary, neurological and dermatological symptoms. While prevention and excellent supportive care are the core tenets of its management, a high suspicion for FES in vulnerable patient cohorts, and early diagnosis leads to better prognosis. A better understanding of the role of the still research-based ‘targeted’ pharmacological therapies, coupled with advances in both the perioperative care of polytraumatised patients and the intra-operative techniques will all help reduce the impact of this condition. Similarly, clearer focus of the impact of FES on the immunomodulation of lung injury will help guide treatment.
